# Obstructed Hemivagina, Uterine Didelphys, and Ipsilateral Renal Agenesis: A Case Report of OHVIRA Syndrome

**DOI:** 10.15388/Amed.2025.32.2.19

**Published:** 2025-12-30

**Authors:** Mehtap Güneş, Sevinç Taşar, Hayriye Nihan Karaman Ayyıldız, İbrahim Kale, Funda Alp Uydu, Murat Muhcu

**Affiliations:** 1 Umraniye Training and Research Hospital, Department of Obstetrics and Gynecology, Istanbul, Turkey; 2 Umraniye Training and Research Hospital, Department of Pediatric Radiology, Istanbul, Turkey; 3 Umraniye Training and Research Hospital, Department of Pediatric Surgery, Istanbul, Turkey; 4 Umraniye Training and Research Hospital, Department of Obstetrics and Gynecology, Istanbul, Turkey; 5 Umraniye Training and Research Hospital, Department of Obstetrics and Gynecology, Istanbul, Turkey; 6 Umraniye Training and Research Hospital, Department of Obstetrics and Gynecology, Maternal Fetal Unit, Istanbul, Turkey

**Keywords:** OHVIRA syndrome, uterus didelphys, hematometrocolpos, renal agenesis, adolescent gyne-cology, OHVIRA sindromas, gimdos didelfija, inkstų agenezė, paauglių ginekologija

## Abstract

**Background:**

Obstructed hemivagina and ipsilateral renal agenesis (OHVIRA) syndrome is a rare congenital anomaly of the female urogenital tract. This report presents the clinical features, diagnostic approach, and management of an adolescent girl with OHVIRA syndrome.

**Clinical case:**

A 14-year-old female was admitted with abdominal pain and irregular menstruation. Ultrasonography and MRI demonstrated uterus didelphys with right-sided hematometrocolpos and ipsilateral renal agenesis. External genital examination revealed an intact hymen with a bluish bulge 2 cm above the hymenal ring. Surgical exploration confirmed an obstructed right hemivagina, which was opened while preserving hymenal integrity. Menstrual blood was drained, and a Foley catheter was placed temporarily to ensure patency. The postoperative course was uneventful, and the patient was discharged on the second day.

**Discussion and Conclusions:**

This case illustrates successful surgical management of OHVIRA syndrome while preserving hymenal integrity. Early diagnosis and imaging are essential to prevent complications such as hematometrocolpos, endometriosis, and infertility.

## Abstract (it)

**Įvadas:**

Obstrukcinės hemimakšties ir ipsilateralinės inkstų agenezės (OHVIRA) sindromas yra reta moterų urogenitalinio trakto įgimta anomalija. Šiame atvejo aprašyme pateikiami klinikiniai šio sindromo požymiai, diagnostikos metodai ir OHVIRA sindromu sergančios paauglės gydymas.

**Klinikinis atvejis:**

14 metų paauglė buvo paguldyta į ligoninę dėl pilvo skausmų ir nereguliarių menstruacijų. Ultragarsinis tyrimas ir MRT parodė, kad paauglei yra dviguba gimda su dešiniosios pusės makšties obstrukcija. Išorinis lytinių organų tyrimas parodė, kad mergystės plėvė yra nepažeista, o 2 cm virš mergystės žiedo yra melsvas iškilimas. Chirurginis tyrimas patvirtino dešinės pusės makšties obstrukciją, išsaugant mergystės plėvės vientisumą. Menstruacinis kraujas buvo išsiurbtas, o *Foley* kateteris įdėtas laikinai, siekiant užtikrinti praeinamumą. Pooperacinis laikotarpis praėjo be komplikacijų, ir pacientė buvo išrašyta antrą dieną.

**Diskusija ir išvados:**

Šis atvejis iliustruoja sėkmingą OHVIRA sindromo chirurginį gydymą, išsaugant mergystės plėvės vientisumą. Ankstyva diagnozė ir vaizdinė diagnostika yra būtina siekiant išvengti komplikacijų, tokių kaip hematometrokolpos, endometriozė ir nevaisingumas.

## Introduction

Obstructed hemivagina and ipsilateral renal agenesis (OHVIRA) syndrome is a rare congenital malformation of the urogenital system. The syndrome is characterized by the presence of a triad consisting of uterus didelphys, a unilateral obstructed hemivagina, and ipsilateral renal agenesis [1]. Although often underestimated due to underdiagnosis and limited awareness, the incidence of OHVIRA syndrome has been reported to range between 0.1% and 3.8% [2]. The exact etiology of OHVIRA syndrome is unknown; however, it is considered to result from a disturbance in the normal embryological development of the female urogenital tract, attributed to abnormal formation of the Müllerian and Wolffian ducts during embryogenesis [2]. The Müllerian ducts give rise to the fallopian tubes, and their remaining portions fuse to form the uterus. Depending on the severity of the fusion defect in the Müllerian ducts, uterine anomalies may occur, ranging from a septum within the uterus to uterus didelphys. The vagina develops from the sinovaginal bulbs, which originate from the distal portions of the Wolffian ducts connected to the urogenital sinus, and the ureteric buds also arise from this region. Consequently, an anomaly in one of the Wolffian ducts can result in an obstructed hemivagina and ipsilateral renal agenesis [3].

Here, we present the clinical features and management of a 14-year-old patient with OHVIRA syndrome who was referred to our institution with a chief complaint of localized abdominal pain.

## Clinical case

**Figure 1:**
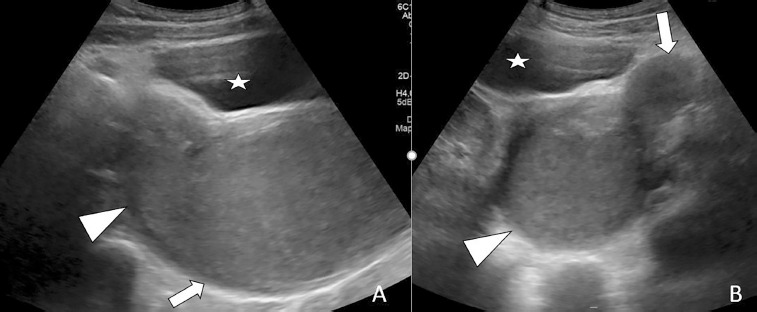
Pelvic ultrasound examination reveals a hematometrocolpos in the sagittal plane, which continues from the vagina (arrow) into the uterine cavity (arrowhead), causing expansion and the appearance of a cystic mass (A). Axial plane images reveal the uterus with a normal endometrial cavity (arrow) adjacent to the uterine and vaginal cavity (arrowhead) filled with hemorrhagic material, consistent with a uterine didelphys variation. Urinary bladder (star).

**Figure 2:**
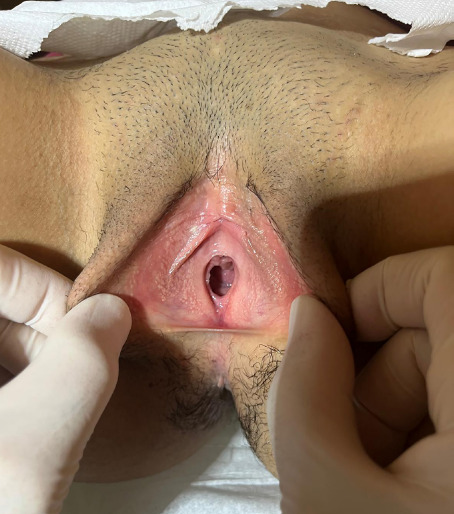
Annular hymen with a normal orifice; the obstructed hemivagina, located approximately 2 cm above the hymenal opening, is not visible in this image.

**Figure 3:**
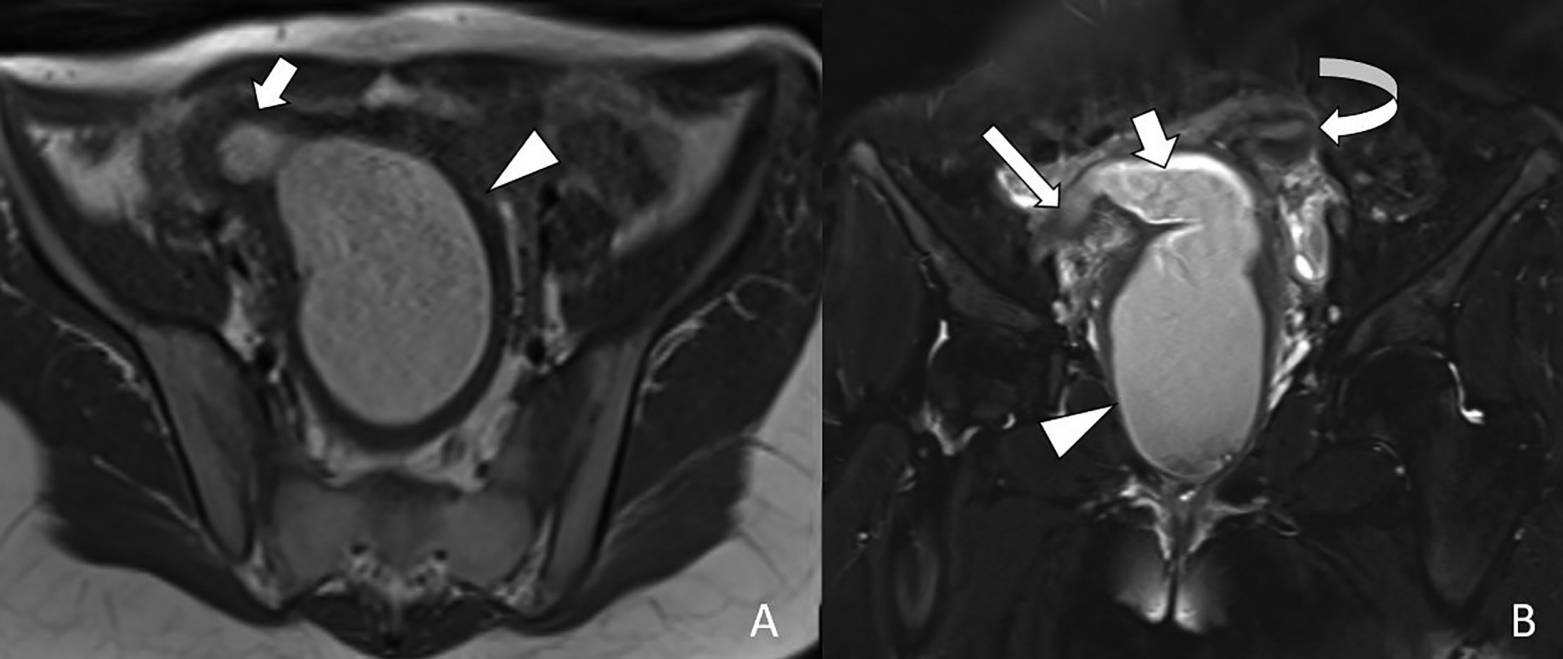
On unenhanced pelvic MRI, axial T1W series show hyperintense signal secondary to hemorrhagic material in the uterine cavity (the short arrow) and vagina (arrowhead) (A). Coronal plane images show a uterine didelphys appearance, and hemorrhagic material is observed in the right-sided uterine cavity (the regular arrow) and vagina (arrowhead). Also, hemorrhagic material continued in the right fallopian tube, causing expansion (the long arrow). The left-sided uterine cavity is normal (the curved arrow), and the vagina is compressed (B).

A 14-year-old adolescent female presented to the emergency department with a two-month history of abdominal pain. The patient reported experiencing irregular menstruation for approximately one year. Suprapubic pelvic ultrasonographic evaluation revealed a normally filled bladder. Imaging was consistent with uterus didelphys. On the right side, a mass measuring 134 × 75 × 73 mm, compatible with hematometrocolpos, was observed. Adjacent to this, the fundus of the contralateral uterus deviated to the left, with a thin, elongated vaginal segment contiguous with hematocolpos. The left kidney appeared normal, whereas the right kidney was not visualized (Figure 1). On external genital examination, the hymen was found to be intact and not imperforate. Approximately 2 cm above the hymenal ring, a bluish-black mucosal bulge was observed protruding into the vaginal lumen on the right side (Figure 2). Subsequently, the patient underwent comprehensive abdominal and pelvic magnetic resonance imaging (MRI). The liver, spleen, gallbladder, pancreas, and both adrenal glands were of normal size and location. The right kidney was not visualized, consistent with agenesis, while the left kidney demonstrated compensatory hypertrophy, measuring 13 × 6 cm. The bladder and both ovaries appeared normal. Imaging confirmed uterus didelphys, with the right uterus containing a mass measuring 132 × 72 × 70 mm, compatible with right hematometrocolpos. Adjacent to this, the contralateral uterus, deviated to the left, demonstrated a normal endometrial cavity. Based on these findings, a diagnosis of OHVIRA syndrome was established (Figure 3).

**Figure 4:**
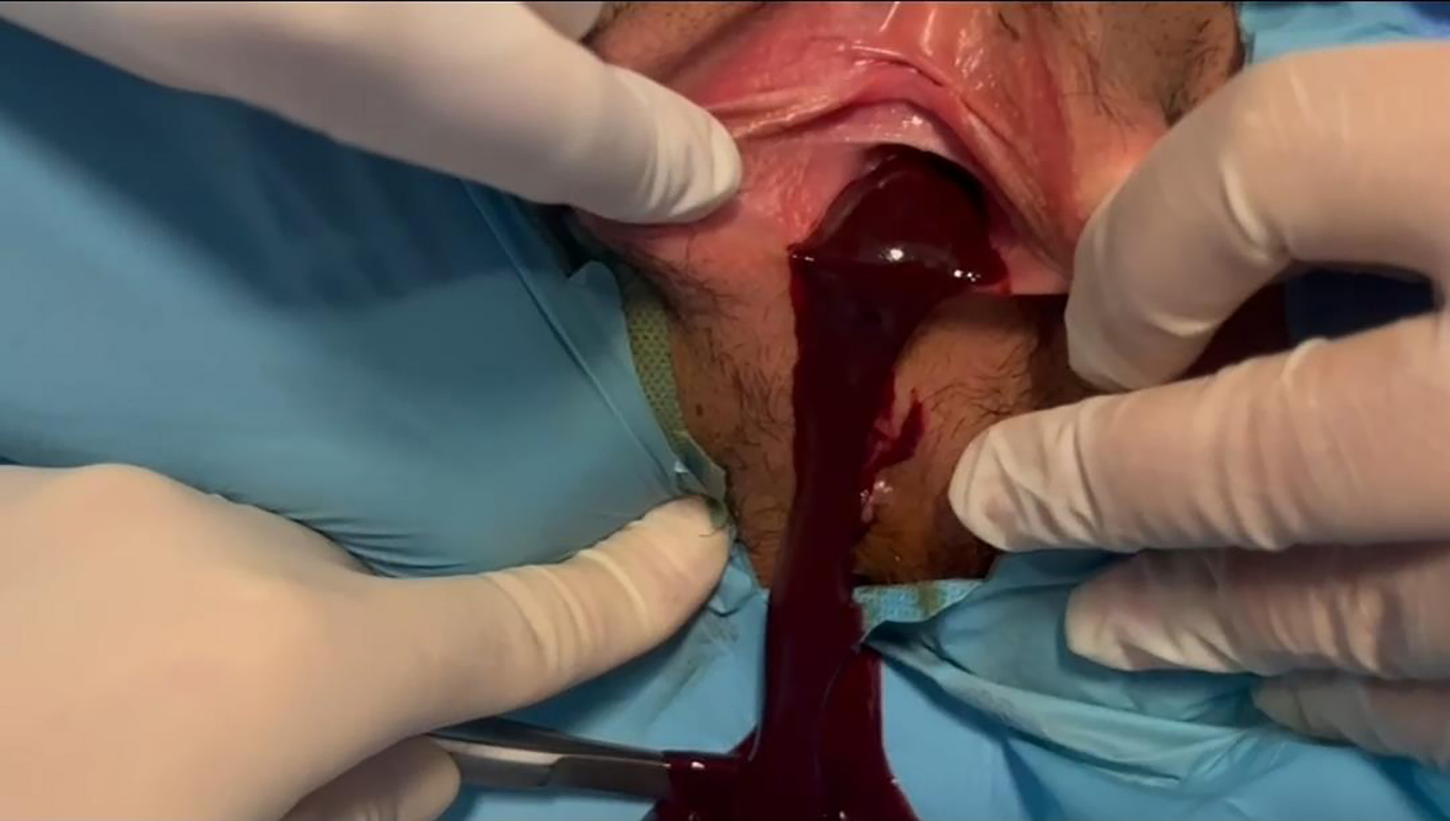
Drainage of retained menstrual blood following the opening of the obstructed hemivagina without disruption of hymenal integrity.

After the patient and her family were informed and written consent was obtained, the patient underwent surgery under general anesthesia. The right obstructed vaginal canal was surgically opened without compromising the hymenal integrity. The accumulated menstrual blood was evacuated (Figure 4). A size 18 Fr Foley catheter was inserted into the vaginal opening to ensure complete drainage and was removed on the first postoperative day. The patient experienced an uneventful recovery and was discharged on the second postoperative day. Written informed consent was obtained from the patient and her legal guardian for the use of the patient’s anonymized medical data and images in this scientific publication.

## Discussion

OHVIRA syndrome, along with Wunderlich and Herlyn-Werner syndromes, comprises a group of anomalies characterized by uterine duplication (didelphic or bicornuate), paracervical cyst formation, various types of vaginal obstruction, and associated renal agenesis [2]. First reported by Herlyn and Werner in 1971, Herlyn-Werner syndrome is defined by the presence of a duplicated uterus, a Gartner’s duct cyst, and ipsilateral renal agenesis [4]. Wunderlich syndrome, first described in 1976, is defined as a connection of the right uterus to the vagina and agenesis of the ipsilateral kidney [5]. OHVIRA syndrome, first documented in 2007, was defined as a spectrum of malformations involving a duplicated uterus and vagina, unilateral vaginal atresia, and absence of the ipsilateral kidney [6].

After the original definitions of these syndromes, reports of cases presenting such anomalies began to appear in the literature. A systematic review published in 2021 evaluated variants of OHVIRA syndrome, encompassing 734 cases across 133 studies. According to this review, the most frequently observed anatomical variants include a left-sided obstructed hemivagina (50.7%), isolated hematocolpos or hydrocolpos (55.9%), uterus didelphys (82.9%), and ipsilateral renal agenesis (92.2%). Vaginal septectomy was the most commonly performed surgical procedure (86.5%). Additional interventions included hemivaginectomy, hemihysterectomy, and total hysterectomy, while some patients required salpingectomy or oophorectomy. In 7.5% of cases, predominantly in infants, surgery was not necessary due to spontaneous resolution of hydrocolpos [2]. In contrast to the most commonly reported variant in this review, our case presented with an obstructed right hemivagina and right-sided hematometrocolpos. The treatment approach we employed involved surgical opening of the obstructed right hemivagina.

In 2025, Oishi and colleagues shared a single-center experience on OHVIRA/Herlyn-Werner-Wunderlich syndrome. A total of nine patients were included, of whom, six were diagnosed with OHVIRA syndrome and three with Herlyn-Werner-Wunderlich syndrome, with a mean age at diagnosis of 20.3 years. Of nine patients, four presented with dysmenorrhea; diagnosis occurred before menarche in three cases, communicating tracts were seen in three, and five underwent surgery or hormone therapy [7].

OHVIRA syndrome is typically diagnosed during adolescence; however, cases initially identified during pregnancy have also been reported. Patients who were followed until term underwent elective cesarean delivery during the third trimester [8–10]. Endometriosis was identified in 13.6% of patients who underwent surgical treatment for OHVIRA syndrome [2]. Different stages of endometriosis and infertility have also been reported during the follow-up after surgical correction of OHVIRA syndrome [11]. Although very rare, cases of gynecological malignancies occurring together with OHVIRA syndrome have also been reported [12–14].

## Conclusions

This case highlights a rare Müllerian anomaly, OHVIRA syndrome, which was successfully managed through surgical opening of the obstructed hemivagina while preserving hymenal integrity. It underscores the importance of considering OHVIRA syndrome in adolescent girls presenting with unexplained abdominal pain and menstrual irregularities, and emphasizes the pivotal role of imaging in achieving an accurate diagnosis. Early recognition is crucial to prevent complications such as hematometrocolpos, endometriosis, infertility, and, albeit rarely, malignancy. Moreover, girls with renal agenesis should be carefully screened for genital tract anomalies, and conversely, those with vaginal or uterine malformations should be evaluated for associated renal abnormalities.
